# Multi- and Transgenerational Histological and Transcriptomic Outcomes of Developmental TCDD Exposure in Zebrafish (*Danio rerio*) Ovary

**DOI:** 10.3390/ijms26146839

**Published:** 2025-07-16

**Authors:** Amelia Paquette, Emma Cavaneau, Alex Haimbaugh, Danielle N. Meyer, Camille Akemann, Nicole Dennis, Tracie R. Baker

**Affiliations:** 1 Department of Environmental and Global Health, University of Florida, Gainesville, FL 32611, USA; amelia.paquette@ufl.edu (A.P.); emmacavaneau4@gmail.com (E.C.); a.haimbaugh@phhp.ufl.edu (A.H.); danielle.meyer@ufl.edu (D.N.M.); n.dennis@phhp.ufl.edu (N.D.); 2 Department of Pharmacology, Wayne State University, Detroit, MI 48202, USA; cakemann14@gmail.com

**Keywords:** TCDD, ovary, atresia, multigenerational, transgenerational, zebrafish

## Abstract

2,3,7,8-Tetrachlorodibenzo-*p*-dioxin (TCDD) exposure has long been associated with reproductive dysfunction in males and females even at miniscule levels, which can persist across generations. Given the continued industrial use and detection of other aryl hydrocarbon receptor (AhR) agonists in the general population and the demonstrated heritable phenotypes of TCDD exposure, further work is justified to elucidate reproductive pathologies and minimize exposure risk. In females, multi- and transgenerational subfertility has been demonstrated in a zebrafish (*Danio rerio*) model exposed to 50 pg/mL TCDD once at 3 and 7 weeks post fertilization (wpf). We further characterize the histopathologic, hormonal and transcriptomic outcomes of the mature female zebrafish ovary following early-life TCDD exposure. Exposure was associated with significantly increased ovarian atresia in the F0 and F1, but not F2 generation. Other oocyte staging and vitellogenesis were unaffected in all generations. Exposed F0 females showed increased levels of whole-body triiodothyronine (T3) and 17β-estradiol (E2) levels, but not vitellogenin (Vtg), 11-ketotestosterone (11-KT), cortisol, thyroxine (T4), or testosterone (T). Ovarian transcriptomics were most dysregulated in the F2. Both F0 and F2, but not F1, showed changes in epigenetic-related gene expression. Rho signaling was the top pathway for both F0 and F2.

## 1. Introduction

As posited by the Developmental Origins of Health and Disease (DOHaD) hypothesis, early life exposure to environmental agents, such as endocrine-disrupting compounds (EDCs), has the potential to interfere with transcriptomic and epigenetic networks during critical windows of development [1]. Such disruption can lead to health effects that present across the lifespan and can be heritable across multiple generations of descendants [2,3,4].

2,3,7,8-Tetrachlorodibenzo-*p*-dioxin (TCDD) occurs both naturally at low levels by natural processes, such as forest fires, and at higher levels as a by-product of plastic waste burning and other manufacturing processes. It is the most potent and toxic dioxin-class EDC, with highly bioaccumulative and lipophilic properties [5]. This model aryl hydrocarbon receptor (AhR) agonist can represent other dioxin-like compounds found in the general population and environment such as polychlorinated biphenyls (PCBs), furans and polycyclic aromatic hydrocarbons (PAHs) [6].

TCDD has well-established effects on multiple organ systems, including cardiovascular [7], immune [8], skeletal [9], neurobehavioral [10] and reproductive systems. Early-life exposure to TCDD is known to alter the reproductive functioning of both males and females, as demonstrated in the outcomes of residential TCDD exposure in Seveso, Italy: adult males exposed before puberty and male offspring of those exposed showed decreased sperm concentration and motility [11,12], while exposed females demonstrated increased infertility, time to pregnancy and risk of endometriosis [13,14]. When modeled in other mammalian species, developmental TCDD exposure resulted in similar reproductive trends, including effects on testes weight and histopathology, reduction in epididymal sperm reserves and sperm motility and dysregulation of steroidogenic and spermatogenic genes in males [15,16,17]. In females, effects included malformed external genitalia, altered ovarian weight and histopathology, premature ovarian failure, reduced levels of estradiol and LH, impaired folliculogenesis and disruption of follicular development and ovulation pathways [18,19,20,21]. In fact, infertile women have been found to carry higher levels of certain dioxin-like compounds in their follicular fluid [22].

Many factors complicate epidemiological studies of the multigenerational consequences of developmental EDC exposure, including confounding exposures, genetic variability, and the time span between exposure and measured outcomes. Zebrafish (*Danio rerio*) are useful in modeling such exposures, due to their quick generation time, ease of exposure, production of numerous, and externally fertilized embryos from the same parents and are additionally well suited to reproductive studies due to their emerging presence as a model of gonadal development [23]. At approximately 3 wpf, zebrafish enter gonadal differentiation, where bipotential tissue resembling an immature ovary either initiates the apoptotic transformation to testes, or progresses to oogenesis [24]. Generally, gonadal fate is determined by approximately 7 wpf, but gonads will continue to develop until reproductive maturity at 3–4 months [23]. These F0 fish are then raised to adulthood and spawned to create an indirectly exposed F1 generation. These F1 fish were likewise spawned as adults to produce the completely unexposed F2 generation, where distinctly transgenerational effects would be observed.

In previous work, TCDD-lineage fish demonstrated impaired reproduction across three generations. Outcrosses between TCDD-lineage and control-lineage fish for all three generations revealed transgenerational reproductive deficits present in TCDD-lineage males [25,26], which our group characterized through histological, transcriptomic and methylomic analyses of F0-F2 testes [27,28,29,30]. In contrast, TCDD-lineage females displayed reproductive deficits into the F0 and F1 generations only, suggesting multigenerational but not transgenerational gonadal disruption. A brief examination of H&E-stained F0-F2 adult female ovaries indicated an increased number of atretic follicles in F0 and F1 TCDD-lineage fish alone, further supporting these multi- but not transgenerational findings [25].

In this study, to robustly characterize the long-term reproductive effects on TCDD-lineage females, we carried out an in-depth histological assessment in F0-F2 ovaries, specifically evaluating ovary size, percentage of atresia, presence of egg debris and gonadal staging [31]. In line with previous work, we found that the percentage of atresia was increased in the F0 and F1, but not F2 ovaries. However, there were no differences for other histological endpoints in the F0 (F1 and F2 unassessed). Interestingly, we found gene expression dysregulation in the F0 and F2 generation, but not the F1, suggesting a transgenerational, but not multigenerational, effect for that outcome. The top pathways in the F0 and F2 generations were both Rho signaling-related, again highlighting the transgenerational link. Some epigenetic-related genes were differentially expressed in the F2 generation, which warrants further investigation. Lastly, we measured hormone levels from whole-body lysate. Exposed females showed increased levels of whole-body triiodothyronine (T3) and, unexpectedly, increased 17β-estradiol (E2) levels. This work gives a fuller picture of the effect of low-level, early TCDD exposure and provides opportunities for future research.

## 2. Results and Discussion

### 2.1. Ovarian Histology

**Figure 1:**
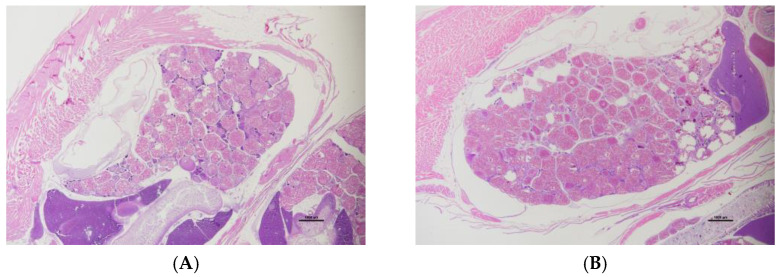
Representative H&E-stained section of F0 DMSO ovary (**A**) and TCDD ovary (**B**). Scale bar is 1000 µm.

OECD-defined gonadal staging [31] was performed on H&E-stained sections of F0 fish. Oocytes in each maturation stage (chromatin nucleolar/perinuclear oocytes, cortical alveolar oocytes, vitellogenic oocytes), atretic follicles, presence/absence of egg debris and granulomatous inflammation were measured (Supplemental Table S1). Representative sections are shown in Figure 1.

Ovary size was not affected in F0 (*p* = 0.687) or F1 (*p* = 0.989) (Student’s two-sample *t*-test). Neither egg debris nor granulomatous inflammation area were affected in F0 (*p* = 0.65 (Fisher’s exact test); *p* = 0.546 (Student’s two-sample *t*-test), respectively), and was not assessed in F1 or F2.

**Figure 2:**
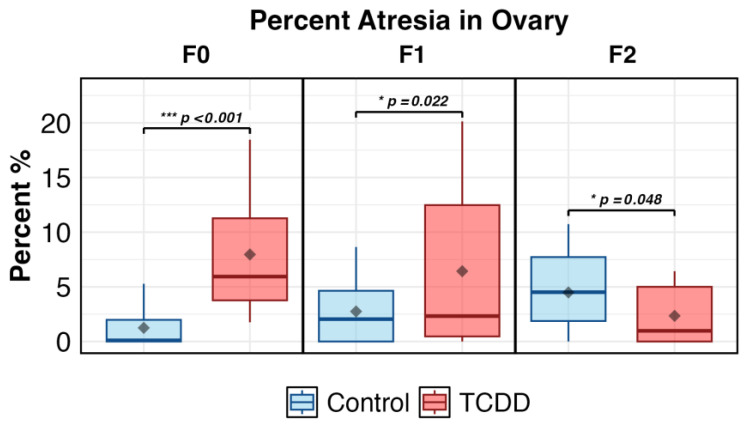
Percentage of atresia in ovary across generations.

Percentage of atretic ovary was increased in the F0 TCDD-exposed fish compared to controls (*p* < 0.001). No other oocyte staging endpoints were affected. The percentage of atresia was the only staging endpoint further assessed in F1 and F2 generations. In the F1, exposure was associated with a significantly higher percentage of atresia (*p* < 0.05); in the F2 there was, unexpectedly, a statistically significant (*p* = 0.048) decrease in atresia compared to controls (Figure 2). This is likely due to the slight increase in control atresia in the F2 compared to F1 and F0, the etiology of which is unknown. While statistically significant, the difference is likely not physiologically relevant in the F2. Similarly, another study found atresia was significantly lower in the F2 exposure group compared to control in TCDD-exposed mice [32], though mice F2 can be considered more comparable to zebrafish F1 [33]. A previous report from this group concurs with the findings of atretic follicles in the F0 and F1, but not F2 generation. Further, spawning failure in the F2 was attributed to male, not female, zebrafish [26]. Thus, we observed a multi- but not trans-generational increase in ovarian atresia following F0 exposure, in line with previous work.

TCDD has been shown to cause atresia in other zebrafish models of TCDD exposure [26,34,35,36,37,38,39]. In rats, number and size of follicles were reduced [40], though mice were unaffected [41]. In mammalian cells, the AhR regulates granulosa cell proliferation [42]. Similar compounds to TCDD such as PAHs have been shown to affect female anovulation, reduced conception, spontaneous abortion, menstrual abnormalities and gonadal developmental defects [43]. Dioxin-like compounds promote the development of endometriosis, a syndrome linked to infertility, pain and irregular menstrual cycles [44,45,46].

Future work will assess gonadal staging in further generations. Though we did not observe altered staging outside of atresia in the F0, TCDD has been associated with decreased mature follicles in zebrafish [35]; multi- and transgenerational effects are yet to be uncovered.

### 2.2. Differentially Expressed Genes and Pathway Analysis

#### 2.2.1. F0 Generation

The directly exposed F0 generation produced 215 differentially expressed genes with ≥1 absolute log_2_ fold change and *p* < 0.01 in the exposed fish (203 upregulated; 12 downregulated; Supplemental Table S2). The highest differential expression was in *klf2b* (Krueppel-like factor 2b) (log2FC = 2.86), orthologous to human KLF2. In zebrafish, this gene encodes a transcription factor involved in germ layer cell fate and embryonic cardiac development [47,48]. In humans, it is a transcriptional activator involved in many biological processes that overlap with TCDD-disrupted processes including adipogenesis [49,50], embryonic erythropoiesis [51,52] and inflammation [53,54].

When DEGs were restricted to those with FDR < 0.1, eight other DEGs remained. These were involved in basic cellular functions such as enzymes (cathepsins *ctsbb* and *degs1*), transporters (*slc7a8a* and *atp1a3b*), a chemokine (*ccl25b*), a transcription factor (*rbm47*) and part of collagen complex (*col12a1a*).

Interestingly, *stm*, which plays a role in egg membrane integrity, ovulation and otolith formation was upregulated (log_2_FC = 2.03, FDR < 0.1). TCDD exposure has been shown to decrease fertility [25,36], shift sex ratios [25,55], disrupt ovarian histopathology [25,37] and disrupt inner ear development [56]. Otoliths, bone-like structures in the inner ear, are crucial in maintaining balance. Previous work found both skeletal malformations and behavioral alterations in adult fish exposed as juveniles to TCDD [25,57]. A future direction would be to examine what, if any, effect ear development has on subsequent behavior and if TCDD-exposed fish behavior shows any similarity.

Due to TCDD’s known multi- and transgenerational inheritance effects, we next compared the 215 DEGs with the EpiFactors Database [58]. Of the 215 DEGs, 191 had known human orthologs. Three upregulated DEGs had matches with the EpiFactors Database: *hdac7*, *ywhae* and *mapkapk3*. These are interesting candidates for further research, as TCDD exposure has known epigenetic effects [59].

Ingenuity Pathway Analysis (IPA) was conducted on 137 analysis-ready molecules of the 215 DEGs uploaded. Table 1 and Table 2 show pathways with z-score cutoff of ≥|2| and *p* < 0.05 for Canonical pathways and Diseases and Functions pathways, respectively. RHO GTP pathways stand out, as do increased cancer pathways. Decreases in inflammation pathways are not unexpected, as TCDD and other AhR ligands may support inflammation or its resolution [53]. These pathways should be considered with caution, as most of the DEGs used for analysis were not significant when considering false discovery rate. Additionally, expected dysregulation of transcripts such as *ahr2*, *cyp1a*, *vtg1* and *star* [55] was not observed. The full IPA Summary can be found in Supplemental Table S4.

#### 2.2.2. F1 Generation

The indirectly exposed F1 generation displayed six DEGs at the level of ≥1 absolute log_2_ fold change and *p* < 0.01; one upregulated (*syne2b*) and five downregulated (*slc35a4*, *trpc4apb*, *cxcr4b*, *stap2b* and one uncharacterized). All of these DEGs additionally met the criteria of FDR-adjusted *p*-value < 0.1. None are known to be related to reproduction. Ingenuity Pathway Analysis was not conducted due to low number of DEGs.

#### 2.2.3. F2 Generation

The unexposed F2 generation exhibited 672 DEGs at the level of ≥1 absolute log_2_ fold change and *p* < 0.01 (556 upregulated; 116 downregulated; Supplemental Table S3. Steroid/Thyroid Hormone LC-MS/MS: Electrospray ionization (ESI) parameters). The F2 produced the most DEGs of any generation. To narrow focus on highly relevant DEGs and pathways, we further filtered DEGs using the FDR-adjusted *p*-value of <0.1. This parameter/filter returned 550 DEGs (454 upregulated; 96 downregulated).

The top upregulated DEG was *rxfp2b* (human ortholog RXFP2) (log_2_FC = 4.96). In fish and mammals, RXFP2 (also known as LGR8) is expressed in gonads [60,61]. In males, its loss of function either genetically or via EDCs has been implicated in cryptorchidism and infertility. When bound by its exclusive ligand INSL3 in the ovary, RXFP2 stimulates androstenedione and thus downstream estrogens production, which then promotes a positive feedback loop with INSL3 [62]. As we later report, we witnessed an increase in E2 hormone levels, though *insl3* expression was not significantly raised (log2FC = 0.768; *p* = 0.160; FDR = NA). Interestingly, in a rat exposure study using brominated flame retardants, which bear a similar chemical structure to TCDD, no effect on *Rxfp2* or *Ahr* was seen in the ovary, though *Insl3* was downregulated [63]. We observed a weakly significant upregulation in *ahr2* (log2FC = 1.140; *p* = 0.015; FDR = 0.161). Unchanged *ahr2* expression would not be surprising as the exposure was singular, brief and in early life two generations ago; slight upregulation indicates long-term changes in xenobiotic response.

The second most upregulated transcript was *lhx1a* (human ortholog LHX1) (log_2_FC = 3.99). In mammals, LHX1 is involved in embryonic urogenital development [64], and LHX1 deletions are candidates for rare congenital syndrome (Mayer-Rokitansky-Küster-Hauser syndrome (MRKH)) involving aplasia of the uterus, vagina and Müllerian ducts [65,66,67]. In zebrafish, there are no known reproductive associations; *lhx1a* functions mainly in renal development [68,69]. The upregulation may be epigenetic and compensatory for the renal effects of ancestral TCDD exposure [39,70], rather than indicative of a genital tract anomaly, especially given the reversal in direction of expression compared to humans with MRKH.

The strong upregulation of *rxfp2b* and *lhx1a* does not fit neatly into existing frameworks of reproductive function. Neither were significantly changed in the F0 generation. Future work would benefit from epigenetic inquiry into this counterintuitive finding.

The most downregulated transcript was *drd4a* (human ortholog DRD4) (log_2_FC = −3.13). *Drd4a* has not yet been shown to express in zebrafish ovary, though it is found in human gonads. In humans, DRD4 encodes the dopamine receptor D4, lower-function variants of which may be implicated in ADHD [71]; likewise, D4 disruption can alter locomotor activity in zebrafish [72,73]. Exposure to AhR agonists including TCDD have been associated with zebrafish hyperactivity [57,74]; this extended to the F1 in one report [57]. Fetal TCDD exposure and AhR overexpression has been shown to activate tyrosine hydroxylase mRNA expression [75], connecting one dot between dopamine and TCDD. It remains to be seen whether *drd4a* has a role mediating this neurobehavioral response, or in reproduction. Behavior and reproduction are quite linked in zebrafish, as both males and females not only must be intrinsically fertile but also perform specific, orchestrated spawning behaviors to release eggs.

The second-most downregulated transcript was *spata22* (human ortholog SPATA22) (log_2_FC = −2.77). SPATA22 is expressed nearly exclusively in germ cells during meiosis and is required for homologous recombination [76,77]. This downregulation is in line with multiple observed ovarian phenotypes. Whole exome sequencing of consanguineous and sporadic cases uncovered loss-of-function SPATA22 variants in primary ovarian insufficiency [78,79]. Diminished reproductive success has been shown in female zebrafish exposed to TCDD [25,35,37]; what, if any, role *spata22* plays in this pathology is unknown. Little, if any, research has been completed on *spata22* in zebrafish. *Spata4*, a testis-specific transcript, was strongly downregulated in one study of TCDD exposure and male zebrafish [28].

Consistent with findings from other transgenerational toxicology models, including fish and rodents models exposed to TCDD and other contaminants and stressors, our results showed an increasing trend in DEG levels in the transgenerational generations compared to the direct exposure effects observed in F0 and F1 generations [28,32,80,81,82]. This pattern demonstrates a compounding effect of with each successive generation. While the underlying mechanisms require further investigation, it has been hypothesized that this phenomenon results from cumulative epigenetic modifications that differ mechanistically between direct exposure and transgenerational inheritance in unexposed descendant generations.

Ingenuity Pathway Analysis (IPA) was conducted for 377 analysis-ready molecules of the 550 DEGs uploaded. With the pathway z-score cutoff of |2|, a theme emerged of disruption of basic cellular functions. The top five up- and downregulated Canonical Pathways are shown in Table 3; Disease and Function pathways in Table 4. In the full dataset under the Categories column in the Top Diseases or Functions list, 43% of terms contain cellular movement, development, signaling or morphology. The full IPA Summary can be found in Supplemental Table S5.

The fact that DEGs were observed multiple generations later led us to compare F2 DEGs with the EpiFactors Database [58]. Of the 550 F2 DEGs, 462 genes had known human orthologs. Of these, there were 21 matches with the EpiFactors Database. These are shown in Table 5. This suggests a demethylated environment, as *tet2* functions in the demethylation process, and *dnmt3b* is a methylase. TCDD is a known epigenetic disruptor [59].

#### 2.2.4. Transgenerational Comparisons

**Figure 3:**
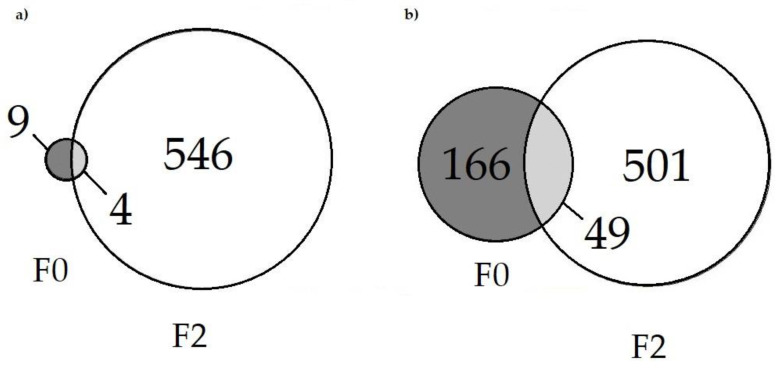
(**a**) F0 and F2 DEGs both at FDR < 0.1 and log2FC > |1. (**b**) DEGs of F0 (*p* < 0.01) and F2 (FDR < 0.1) at log2FC > |1|.

F2 generation had some overlap in DEGs with the F0 generation (Figure 3). With parameters set at log2FC > |1| and FDR < 0.1, there were only 4 DEGs (*ccl25b*, *atp1a3b*, *degs1* and *col12a1a*). An exploratory comparison using the more promiscuous cutoff of *p* < 0.01 for the F0 produced a similar ratio of overlap (approximately one third of the DEGs of the F0 overlapped with F2 DEGs).

Remarkably, the top up- and downregulated pathway in both the F0 and F2 IPA were Rho GTPase cycle and Rho GDI signaling, respectively, with neutrophil degranulation being the second most upregulated pathway in both generations as well; despite the more promiscuous F0 *p*-value cutoff to allow enough DEGs for pathway analysis (*p* < 0.01 vs. FDR < 0.1).

IPA was also run with the overlapping F0 and F2 DEGs from Figure 3b. Of the 49 DEGs uploaded, 37 were mapped. All 37 molecules were involved in the significant pathways of Organismal Injury and Abnormality and Cancer. These results are taken with skepticism, as Qiagen’s training materials recommend the minimum number of molecules for analysis be 200 [83], and directionality of expression in either generation was not considered, though TCDD exposure has been associated with abnormal morphology [84] and cancer [85].

The only overlap in EpiFactor Database genes between F0 and F2 is *ywhae2*, upregulated in both generations. This gene is an excellent candidate for future scoping epigenetic studies in TCDD exposure in the zebrafish ovary. Additionally, alterations in YWHAE have been observed in human gynecological sarcomas [86].

We observed differential gene expression two generations out from a brief early-life exposure, with negligible change in the F1 generation. The transgenerational gene expression and pathway findings are ample fodder for pursuing epigenetic analyses such as differential methylation in the TCDD-exposed ovary. Supporting this direction, our group previously found multi- and transgenerational methylome changes in adult testes exposed to the same TCDD paradigm [30]. The epigenetic landscape of the ancestrally TCDD-exposed ovary is to be explored.

### 2.3. Hormone Analysis

**Figure 4:**
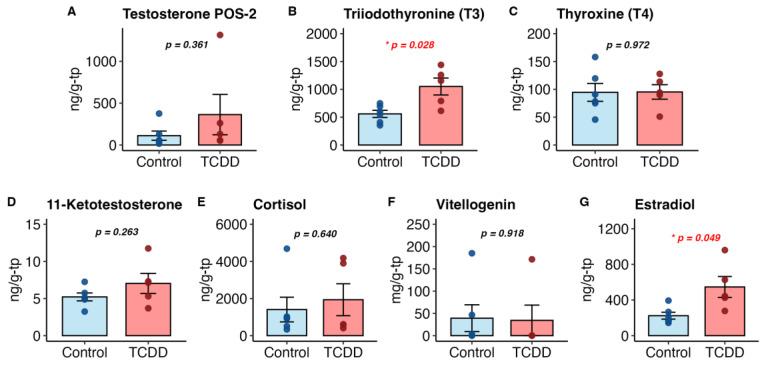
Boxplots of whole-body mean hormone level changes following exposure in the F0 generation. Welch’s *t*-test. T: testosterone. T4: thyroxine. T3: triiodothyronine. 11-KT: 11-ketotestosterone. E2: 17β-estradiol. Vtg: vitellogenin.

The F0 generation was examined for differences in hormone levels. The ovary expresses AhR and androgen [42,87], and TCDD exposure has been shown to disrupt ovarian steroidogenesis [88]. Whole-body lysates contained significantly higher levels of triiodothyronine (T3) (*p* = 0.028) and 17β-estradiol (E2) (*p* = 0.049) in TCDD-exposed female fish as compared to DMSO controls. Testosterone (T), thyroxine (T4), 11-ketotestosterone (11-KT) and vitellogenin (Vtg) levels were not significantly affected (Figure 4).

Other species have mixed thyroid hormone responses to TCDD exposure. Increased T3 following chronic exposure was observed in female rats [89]; in vitro chicken thyroid cells exhibited significantly decreased T3 secretion [90]; and in women following a large explosion of a dioxin factory, there was no association with T3 [91]. Men exposed as agricultural workers displayed lowered T3 [92], though no difference was observed in another epidemiological study on males exposed as veterans for T3, or for T4 [93]. Downward trends in T4 are often observed in multiple models [90,91,94,95,96], though we did not observe T4 changes.

Unexpectedly, we observed increased 17β-estradiol (E2) levels with TCDD exposure. Generally, E2 is lowered in response to TCDD, including in female zebrafish [19,35,41,97,98,99,100,101]). However, response may also vary by cell type: a single TCDD exposure increased E2 secretion overall in porcine preovulatory follicles, including in theca cells, but decreased secretion in granulosa cells [102], while some find no effect [103].

Though we did not find changes in Vtg, T, 11-KT, or cortisol, others have shown TCDD-induced disruption in one or more of these. Vtg, an egg yolk precursor protein, is a reliably lowered marker following TCDD exposure [35,98,104,105], though our results do not reflect a significant change in Vtg levels. Testosterone is also reliably decreased in multiple models following TCDD exposure [41,92,102,106]. Curiously, our data, while insignificantly different between controls and TCDD, show a rising trend in T with exposure, and one exposed fish was a high outlier. High T has been observed in women seeking IVF treatment; compared to low-T cases these women had significant decreases in oocyte and embryo quality, fertilization and cleavage rates and upregulated *Ahr* RNA, despite no direct TCDD exposure [107].

11-KT and cortisol are both important in teleost for oocyte development, and in some species 11-KT is the most potent male androgen [108,109]. We saw no difference in 11-KT levels and a statistically insignificant increase in cortisol following exposure. Very little research has been conducted into the relationship between 11-KT and reproductive outcomes in zebrafish. In a knockout model of the enzyme that biosynthesizes both 11-KT and cortisol (11β-hydroxylase), zebrafish with lowered levels of 11-KT and cortisol had smaller genital papilla and decreased oocyte maturation [109]. AhR agonists can also disrupt expected cortisol levels in response to stress [110].

The lack of increased hormonal and thyroid levels and unexpected results may be explained by the extended assessment timeframe (>10 months post-exposure). TCDD exposure likely disrupts HPA-HPG axis function most acutely during or immediately following exposure, as most studies document hormonal changes during early development, ongoing exposure, or acute post-exposure periods in adults. Early developmental hormonal alterations may trigger lasting structural and epigenetic changes in endocrine and reproductive tissues, causing later reproductive dysfunction. Future longitudinal hormone assessments spanning juvenile development will test this hypothesis.

Also, hormones may not be the most sensitive or reliable endpoint to study reproductive health in female zebrafish. Despite this, future work from this lab will examine hormones in the F1 and F2 generations. It is known that certain steroid hormones are passed from female fish to their yolk [111], and further, some TCDD body burden is maternally transferred to F1 embryos [36]. In women exposed to TCDD, altered T3 levels did not manifest until later in their lineage, where maternal serum TCDD was associated with lower T3 in offspring [112]. It is worthwhile to continue to examine hormone changes across generations, and may be beneficial to increase the *n*-value (and thus tissue mass) to where the ovary can be assessed specifically, rather than whole-body lysate.

## 3. Materials and Methods

### 3.1. Fish Husbandry

Fish husbandry for the endpoints of histology and gene expression analysis proceeded as reported in [28]; WSU and UWM IACUC Protocol No. M00489. A separate cohort of fish was raised for hormone analysis at Wayne State University according to the ethical standards of the National Institutes of Health Guide for Care and Use of Laboratory Animals. All protocols were approved by the Wayne State University Institutional Animal Care and Use Committee (protocol number 19-02-0938; approved 14 October 2020).

### 3.2. Exposure

These methods were reported previously in [27]. Briefly, TCDD (>99% purity) (Chemsyn; Concord, ON, Canada) was dissolved in DMSO. The F0 generation of zebrafish were exposed for 1 h to either 50 pg/mL TCDD or 0.1% DMSO control during sexual differentiation and maturation at 3 and 7 weeks post fertilization (wpf). At 6 months of age, the F0 fish were spawned to create the F1 generation. These fish were indirectly exposed as germ cells within the F0 generation. The F1 fish were also spawned at 6 months to produce the F2 generation. These fish were never exposed directly or indirectly. F0–F2 female fish were euthanized in tricaine for analysis at 12 mpf.

### 3.3. Ovarian Histology

These methods were reported previously in [27]. Briefly, euthanized fish were formalin-fixed, sagittally bisected, paraffin-embedded, sectioned at 5 μm, mounted on slides and stained with H&E. Zebrafish ovaries were histologically analyzed for atresia in all three generations: F0 (*n* = 10 fish/condition), F1 (*n* = 15 fish/condition) and F2 (*n* = 11 DMSO; 13 TCDD). F0 fish were additionally assessed for gonadal maturation staging. Using a Nikon SMZ18 stereomicroscope and a Nikon DS-Qi2 camera (Nikon; Melville, NY, USA), pictures were taken of each side (A/B) of the bisected ovary per fish.

### 3.4. Histopathological Endpoints

Ovarian maturation staging and histopathological endpoints followed the OECD Guidance Document for the Diagnosis of Endocrine-Related Histopathology of Fish Gonads [31]. The assessed OECD maturation stages include oogonia, chromatin nuclear oocytes, perinuclear oocytes, cortical alveolar oocytes, early vitellogenic oocytes and mature/spawning oocytes. We grouped by maturation stages as previously reported in [25]: small primary follicles (oogonia where visible, chromatin nuclear oocytes/ perinuclear oocytes), intermediate secondary follicles (cortical alveolar oocytes) and large tertiary follicles (vitellogenic oocytes, including mature/spawning). Additionally, histopathologic atresia (degradation and absorption of oocyte/clumping or perforation of the chorion and disorganized ooplasm), granulomatous inflammation (granulomas from increased epithelioid macrophages) and egg debris (extra-chorionic homogenous yolk material) were quantified.

Using ImageJ v.1.53t [113], the number and area (μm^2^) of oocytes in each maturation stage, atresia area, granulomatous inflammation area, the presence of egg debris, whole ovary area and the number of atretic cells were quantified. Significance was determined using Welch’s *t*-test.

### 3.5. RNA Isolation

From each generation (F0, F1 and F2) of fish, ten (TCDD *n* = 5; DMSO *n* = 5) were euthanized. Ovarian tissue was extracted, flash-frozen in liquid nitrogen and stored at −80 °C. Tissue was homogenized in QIAzol lysis reagent (Qiagen, Germantown, MD, USA), and RNA was isolated with the Qiagen RNEasy mini kit. RNA was quantified with Qubit 3.0 Fluorometer (Invitrogen, Carlsbad, CA, USA). The Agilent RNA 6000 Nano kit was used to assess RNA quality using an Agilent Bioanalyzer (Agilent, Santa Clara, CA, USA).

### 3.6. Differential Expression Analysis

Briefly, 3′ mRNA-seq libraries were prepared from isolated RNA using manufacturer’s instructions from the QuantSeq 3′ mRNA-Seq Library Prep Kit FWD for Illumina (Lexogen, Vienna, Austria). Samples were normalized to 40 ng/μL (total input of 200 ng in 5 μL) and amplified at 17 cycles. Libraries were quantified using a Qubit 3.0 Fluorometer and Qubit^®^ dsDNA Broad Range Assay Kit (Invitrogen, Carlsbad, CA, USA), and run on an Agilent TapeStation 2200 (Agilent Technologies, Santa Clara, CA, USA) for quality control. The samples were sequenced on a HiSeq 2500 (Illumina, San Diego, CA, USA) in rapid mode (single-end 50 bp reads). Reads were aligned to *D. rerio* (genome assembly GRCz11 (danRer11)) using the BlueBee Genomics Platform (BlueBee, Rijswijk, The Netherlands). One of the DMSO samples from the F2 generation was identified as an outlier due to low number of total reads and thus dropped from analysis. Lexogen performed in-house DESeq2 analysis on the remaining four F2 samples.

### 3.7. Pathway Analysis

The Ingenuity Pathway Analysis (IPA) software, version 2022.4 (Qiagen, Germantown, MD, USA) was used to generate functional pathways. Significant differential expression was defined by a log2fold change of ≥1 or ≤−1 and a false discovery rate (FDR) adjusted *p*-value < 0.1. Using Ensemble IDs as identifiers, the differentially expressed genes (DEGs) meeting these criteria were uploaded into IPA.

### 3.8. Hormone Analysis

#### 3.8.1. Sample Homogenization

At 12 months post-fertilization (mpf) (+/− 2 weeks), adult female F0 TCDD (n = 6)- and DMSO (n = 6)-exposed fish were ice euthanized, flash frozen in liquid nitrogen and kept at –80 °C until processed. The samples were sent to the Analytical Toxicology Core Laboratory (ATCL) in the Center for Environmental and Human Toxicology at the University of Florida for hormone analysis (Gainesville, FL, USA). The hormones and proteins targeted for analysis were vitellogenin, cortisol, 11-ketotestosterone (11-KT), 17β-estradiol (E2), testosterone, triiodothyronine (T3) and thyroxine (T4). Due to the impracticality of sampling plasma from zebrafish for hormone analysis, a surrogate blood plasma matrix was created from the flash frozen whole fish samples for extraction. Samples were removed from the freezer and dipped in liquid nitrogen for a 15 s hard freeze, then placed between folded wax paper and crushed with a pestle. Crushed tissue pieces were refrozen with a second dip in liquid nitrogen. Tissue was vortexed with 100 μL of PBS for 10 min to homogenize in 2 mL Eppendorf tubes, then centrifuged at 14,000 rpm for 10 min at 4 °C. Liquid slurry was transferred to fresh 2 mL tubes, then 25 μL aliquots were removed for total protein and vitellogenin analysis and placed at −20 °C. Total protein was obtained using a standard Coomassie Blue spectral assay (Thermo Fisher Scientific, Waltham, MA, USA). The remainder of liquid slurry was processed for steroid/thyroid hormone analysis.

#### 3.8.2. Vitellogenin ELISA Assay

Vitellogenin (Vtg) in the whole-body homogenate was measured using a sandwich enzyme-linked immunosorbent assay (ELISA) according to the manufacturer’s instructions [Zebrafish (*Danio rerio*) vitellogenin ELISA kit (V01008402; Biosense Laboratories AS; Bergen, Norway)]. Briefly, 100 μL of diluted Vtg standards and samples (1:500; 1:2500; 1:10,000; 1:50,000; 1:250,000) were added in duplicate to the wells and plates were incubated at room temperature for 60 min. Plates were washed three times with 300 μL washing buffer per well and 100 μL of 1:350 diluted Vtg-specific detecting antibody was added to all wells, followed by a second 60 min incubation at room temperature. Plates were then washed three times as above, and 100 μL of 1:2000 diluted enzyme-labeled secondary antibody was added to all wells. Plates were incubated again as above. Finally, plates were washed five times as above, and 100 μL OPD-peroxidase substrate solution was added to all wells. Plates were incubated in the dark at room temperature for 30 min, after which 50 μL of 2M H_2_SO_4_ were added to the wells to stop the reaction. Vitellogenin was detected within 5 min of adding the stop solution, and the absorbance (OD) of each well was measured at 492 nm with a microtiter plate reader. After correcting standard and sample absorbance values for NSB, sample Vtg concentrations were calculated from the standard curve generated from the standard dilutions and normalized to mg total protein.

#### 3.8.3. Steroid/Thyroid Hormone Assay

To the remaining extract of each sample, 100 μL of 2× anti-oxidant solution (50 mg dithiothreitol (DTT), 50 mg L-ascorbic acid and 50 mg citric acid in 1 mL Optima^®^ water), and 300 μL PBS were auto vortexed for 10 min, then centrifuged at 14,000 rpm for 10 min at 4 °C. Samples were spiked with 10 μL of 50ng/mL steroid/thyroid hormone internal standards mixture in methanol (Estradiol-d5, cortisol-d7, test-d3, 13C6-T3 and 13C6-T4; 50 ng/mL), and 1 mL of chilled MTBE was added, and samples were vortexed for 20 s, then centrifuged at 14,000 rpm for 10 min at 4 °C. The top organic layer was transferred to glass vials, then samples were evaporated and re-constituted 100 μL 1:1 H_2_O:MeOH vortexed for 20 s, transferred to clean 2 mL centrifuge tubes and centrifuged at 14,000 rpm for 20 min at 4 °C. The supernatant was transferred to LC vials with spring inserts and analyzed immediately or stored at −20 °C.

Residue values were obtained using a Shimadzu Nexera X2 Ultra-high-performance liquid chromatograph (UHPLC, Shimadzu Co., Kyoto, Japan) coupled to a triple quadrupole linear ion trap (Qtrap 6500, AB Sciex, Framingham, MA, USA). Data was acquired using scheduled multiple reaction monitoring (sMRM) in both positive and negative polarity modes. Analyte separation was achieved using a Kinetix PS-C18 column (2.1 mm × 150 mm, 2.6 µm i.d., Phenomenex, Torrance, CA, USA) coupled with the associated guard column. A binary gradient of 0.2 mM ammonium fluoride (NH4F) in water as mobile phase A and 100% MeOH as mobile B was utilized starting at 50% mobile phase A and B. The flow rate was 0.25 mL/min, and the injection volume was 8 µL. The total run time for each sample was 15.2 min including equilibration between acquisitions. A 10-point calibration curve (0.01 to 200 ng/mL) was used for analyte quantification. A solvent blank, continuing calibration check standard (CCCS) of known concentration, and another solvent blank ran after every 10 sample injections to ensure calibrated results and to monitor for carryover. Details regarding instrumental limits and analysis and analyte quantification are provided in Supplemental Tables S6–S9. Residue values were provided with and without normalization to total protein. Sample AB4-TF2 was diluted 1:5 as cortisol levels were above the calibration range of 200 ng/mL.

#### 3.8.4. Statistics

Welch’s *t*-test was performed for all hormone comparisons.

## Figures and Tables

**Table 1:** Top Canonical significant pathways for F0.

Ingenuity Canonical Pathways	−log(*p*-Value)	z-Score
RHO GTPase cycle	3.96	3.16
Neutrophil degranulation	4.45	2.71
Molecular Mechanisms of Cancer	1.88	2.53
Post-translational protein phosphorylation	4.72	2.45
Regulation of Insulin-like Growth Factor (IGF) transport and uptake by IGFBPs	4.36	2.45
RHOGDI Signaling	3.85	−2.45

−log(0.05) = 1.3.

**Table 2:** Top Diseases and Functions significant pathways for F0.

Categories	Functions	Diseases or Functions Annotation	*p*-Value	Bias-Corrected z-Score
Cancer, Organismal Injury and Abnormalities	cancer	Cancer of cells	5.33 × 10^−11^	2.154
Cancer, Organismal Injury and Abnormalities, Respiratory Disease	lung tumor	Lung tumor	2.32 × 10^−14^	2.021
Inflammatory Disease, Inflammatory Response, Organismal Injury and Abnormalities, Respiratory Disease	inflammation	Inflammation of lung	0.000121	−2.068
Cell Death and Survival, Organismal Injury and Abnormalities	apoptosis	Apoptosis of carcinoma cell lines	6.21 × 10^−9^	−2.14
Cardiovascular Disease, Organismal Injury and Abnormalities	size	Size of infarct	0.00000019	−2.298
Inflammatory Response, Organismal Injury and Abnormalities, Respiratory Disease	inflammation	Inflammation of respiratory system component	0.000000239	−2.326

**Table 3:** Top Canonical significant pathways for F2.

Ingenuity Canonical Pathways	−log(*p*-Value)	z-Score
RHO GTPase cycle	5.14	4.47
Neutrophil degranulation	3.31	4.12
Phagosome Formation	2.24	3.44
Integrin Signaling	6.47	3.36
Actin Cytoskeleton Signaling	6.41	3.32
Heme signaling	3.29	−2
Mitochondrial Dysfunction	2.87	−2.31
Granzyme A Signaling	3.17	−2.45
RHOGDI Signaling	6.97	−2.53

**Table 4:** Top Disease and Function significant pathways for F2.

Categories	Functions	Diseases or Functions Annotation	*p*-Value	Bias-Corrected z-Score
Cellular Movement	cell movement	Cell movement	2.73 × 10^−23^	2.30
Cell-To-Cell Signaling and Interaction, Inflammatory Response	immune response	Immune response of leukocytes	0.000000424	2.12
Cell Morphology	sprouting	Sprouting	1.37 × 10^−14^	2.11
Cellular Movement	invasion	Invasion of cells	2.41 × 10^−19^	2.09
Cell-To-Cell Signaling and Interaction, Hematological System Development and Function	activation	Activation of myeloid cells	5.64 × 10^−10^	2.08
Cardiovascular Disease, Organismal Injury and Abnormalities	infarction	Infarction	1.82 × 10^−11^	−2.04
Cell Death and Survival, Organismal Injury and Abnormalities	apoptosis	Apoptosis of tumor cell lines	5.3 × 10^−10^	−2.06
Cancer, Organismal Injury and Abnormalities	incidence	Incidence of tumor	2.03 × 10^−26^	−2.09
Cancer, Gastrointestinal Disease, Organismal Injury and Abnormalities	colon tumor	Colon tumor	1.75 × 10^−16^	−2.17
Cancer, Gastrointestinal Disease, Organismal Injury and Abnormalities	colorectal neoplasia	Colorectal tumor	1.32 × 10^−17^	−2.21

**Table 5:** Matches between F2 DEGs and the Epifactors database.

Zebrafish Gene ID	Human Ortholog	Log2FC
*tet2*	TET2	1.86
*chd3*	CHD3	1.71
*sfmbt1*	SFMBT1	1.68
*celf3a*	CELF3	1.63
*phip*	PHIP	1.61
*chd5*	CHD5	1.56
*ywhae2*	YWHAE	1.56
*zgpat*	ZGPAT	1.43
*zmynd8*	ZMYND8	1.39
*foxp4*	FOXP4	1.32
*prdm11*	PRDM11	1.29
*top2b*	TOP2B	1.26
*srsf1b*	SRSF1	1.14
*kmt2e*	KMT2E	1.13
*smarcad1b*	SMARCAD1	1.11
*cbx6*	CBX6	1.10
*foxo1a*	FOXO1	1.04
*srsf10b*	SRSF10	−1.11
*ubr7*	UBR7	−1.19
*taf4a*	TAF4	−1.43
*dnmt3bb.1*	DNMT3B	−1.46

## Data Availability

Data is contained within the article and Supplementary Material.

## Supplementary Materials

The following supporting information can be downloaded at: https://www.mdpi.com/article/10.3390/ijms26146839/s1.
