# Supplementary material for: Multi- and Transgenerational Histological and Transcriptomic Outcomes of Developmental TCDD Exposure in Zebrafish (Danio rerio) Ovary
Source: Int J Mol Sci. 2025 Jul 16;26(14):6839. doi: 10.3390/ijms26146839 (PMC12296038; doi:10.3390/ijms26146839)
Supplement: Supplementary file 1 [file ijms-26-06839-s001.zip › ijms-3614266_Supplemental Hormone date_Tables S6-S9.pdf]

<u>Supplemental Table S6.</u> Steroid/Thyroid Hormone LC-MS/MS: Lower limit of quantification (LLOQ) values for the instrument and for the sample sets.

Hormone	Instrument LLOQ (ng/mL)	Sample LLOQ (pg/mg)
Cortisol	0.01	0.43
11-ketotestoterone (11-KT)	0.01	0.43
17β-estradiol (E2)	0.10	4.30
Testosterone	0.01	0.43
Triiodothyronine (T3)	0.01	0.43
Thyroxine (T4)	0.01	0.43

Protein average (23.1 mg/mL) was used to calculate the sample LLOQ according to the following:

$$Sample\ LOD\left(\frac{\text{pg}}{\text{mg}}\right) = \frac{\left[\text{Instrument LLOQ }\left(\frac{ng}{mL}\right)\right] * 1000}{\text{protein avg. of 23.1 }\left(\frac{mg}{mL}\right)}$$

<u>Supplemental Table S7.</u> Steroid/Thyroid Hormone LC-MS/MS: The solvent gradient used for chromatographic separation in terms of percent mobile phase B (100% MeOH).

Polarity Gradient (%B)
Initial: 50
0.2 min: 65
10.0 min: 70
10.2 min: 99
13.7 min: 99
14.2 min: 50
15.2 min: 50

Supplemental Table S8. Steroid/Thyroid Hormone LC-MS/MS: Multiple reaction monitoring (MRM) polarity (+,-), mass transitions (Q1, Q2), retention times (RT), analyte transition names (ID), declustering potential (DP), entrance potential (EP), collision energy (CE), and cell exit potential (CXP) for data acquisition.

Polarity	Q1	Q3	RT	ID	DP	EP	CE	CXP
+	363.1	121.1	3.14	Cortisol 1	80	10	32	9
+	363.1	309	3.14	Cortisol 2 (quant)	80	10	24	18
+	370.2	125	3.17	Cortisol-d7 1	85	10	32	12
+	370.2	316	3.17	Cortisol-d7 2	85	10	25	22
+	303.2	121	3.31	11-ketotestosterone 1	60	10	34	12
+	303.2	259	3.31	11-ketotestosterone 2 (quant)	60	10	32	12
+	289.2	97	5.61	Testosterone 1 (quant)	100	10	27	12
+	289.2	109	5.61	Testosterone 2	100	10	30	12
+	292.2	97	5.57	Testosterone-d3 1	100	10	27	12
+	292.2	109	5.57	Testosterone-d3 2	100	10	27	12
+	777.7	731.7	4.1	Thyroxine (T4) 1 (quant)	80	10	35	15
+	777.7	633.7	4.1	Thyroxine (T4) 2	80	10	35	15
+	783.7	737.9	4.1	$^{13}C_6$-T4 1	80	10	35	15
+	783.7	639.7	4.1	$^{13}C_6$-T4 2	80	10	35	15
+	651.8	605.8	3.15	Triiodothyronine (T3) 1 (quant)	80	10	26	15
+	651.8	507.8	3.15	Triiodothyronine (T3) 2	80	10	26	15
+	657.67	611.7	3.15	$^{13}C_6$-T3 1	80	10	26	15
+	657.67	513.9	3.15	$^{13}C_6$-T3 2	80	10	26	15
-	271.2	183.1	4.72	17β-estradiol (E2) 1	-140	-10	-49	-18
-	271.2	145.1	4.72	17β-estradiol (E2) 2 (quant)	-140	-10	-52	-22
-	276.2	187	4.67	E2-d5 1	-120	-10	-52	-7
-	276.2	147	4.67	E2-d5 2	-120	-10	-52	-8

Supplemental Table S9. Steroid/Thyroid Hormone LC-MS/MS: Electrospray ionization (ESI) parameters used for positive and negative mode data acquisition.

ESI parameters	Positive mode	Negative mode
Curtain gas (L/Hr)	35	25
Collision gas (L/Hr)	12	12
Ion Spray voltage (V)	5250	-4400
Temperature (°C)	550	550
Ion source gas 1 (L/Hr)	60	35
Ion source gas 2 (L/Hr)	50	50
