# Supplementary material for: Multi- and Transgenerational Histological and Transcriptomic Outcomes of Developmental TCDD Exposure in Zebrafish (Danio rerio) Ovary
Source: Int J Mol Sci. 2025 Jul 16;26(14):6839. doi: 10.3390/ijms26146839 (PMC12296038; doi:10.3390/ijms26146839)
Supplement: Supplementary file 1 [file ijms-26-06839-s001.zip › ijms-3614266_Table S1.pdf]

Supplemental Table S1. F0 gonadal staging and other histology results. Student's t-test unless otherwise noted. N=7 fish per condition; 4* slides per fish. Color corresponds to staging boundaries in Figure 1. * 3 slides for 1 of 7 TCDD fish due to fixation artifact. ° Fisher's exact test.

Stage	Chromatin nucleolar/perinucleolar oocytes- orange		Cortical alveolar oocytes- pink		Vitellogenic oocytes- yellow		Atretic follicles- dark gray		Granulomatous inflammation area- black	
Exposure	DMSO	TCDD	DMSO	TCDD	DMSO	TCDD	DMSO	TCDD	DMSO	TCDD
Mean number	37.357	50.78	16.25	16.22	20.61	19.52	8.82	10.22	N/A	N/A
Standard deviation (number)	20.97	36.27	7.50	7.13	7.91	6.68	6.72	7.33	N/A	N/A
Mean of area (µm)	95003.24	112112.03	441531.53	453823.53	2825526.27	2815948.42	405047.67	675183.36	330331.71	367741.95
Standard deviation (area)	68725.25	96910.61	256859.69	254370.16	687327.61	1032869.70	369054.82	831033.51	158804.63	278397.30
p-value, number	0.102		0.989		0.583		0.464		N/A	
p-value, area (µm)	0.455		0.859		0.968		0.124		0.546	

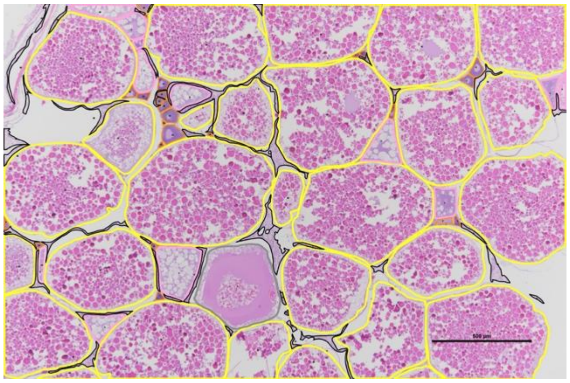

Representative image of ovary cell staging annotation (colors correspond with table above)

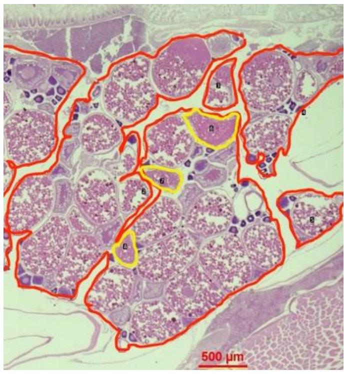

Representative image and ovary area annotation (Red outline- ovary area; yellow outline- atresia area)

Endpoint	Ovary area	
Exposure	DMSO	TCDD
Mean number	N/A	N/A
Standard deviation (number)	N/A	N/A
Mean of area (µm)	30993719.24	28643414.55
Standard deviation (area)	9529100.90	15064437
p-value, area (µm)	0.622	

Endpoint	Atresia in ovary	
Exposure	DMSO	TCDD
Mean percent	2.80	11.55
Standard deviation (percent)	4.44	8.74
Mean area (µm)	28385112.08	28643414.54
Standard deviation (area)	1954876.79	2268953.46
p-value, percent	**0.000576**	
p-value, area (µm)	0.099	

Endpoint	Egg debris (presence: Yes/No)	
Exposure	DMSO	TCDD
Yes	5	5
No	7	3
Total	10	10
p-value	0.65 °	
